# Genome-Wide DNA Methylation and Transcriptome Analyses Reveal Epigenetic and Genetic Mechanisms Underlying Sex Maintenance of Adult Chinese Alligator

**DOI:** 10.3389/fgene.2021.655900

**Published:** 2021-03-11

**Authors:** Jian-Qing Lin, Jun Yu, Li Sun, Sheng-Guo Fang

**Affiliations:** MOE Key Laboratory of Biosystems Homeostasis and Protection, State Conservation Centre for Gene Resources of Endangered Wildlife, College of Life Sciences, Zhejiang University, Hangzhou, China

**Keywords:** Chinese alligator, DNA methylation, transcriptome, sex maintenance, epigenetic

## Abstract

The sexes of Chinese alligators are determined during embryonic development and remain fixed thereafter. In this study, we investigated the genetic and epigenetic mechanisms underlying sex maintenance in Chinese alligators through RNA sequencing and bisulfite sequencing data analyses of the adult gonads. We identified the genes and pathways (e. g., DMRT1-SOX9-AMH pathway for males and oocyte meiotic maturation pathway for females) involved in male and female sex maintenance and gonadal development of adult Chinese alligators. In contrast to their expression patterns in the embryo, both *DMRT1* and the steroid hormone biosynthesis related genes showed a male-biased expression in adult gonads. The overall DNA methylation density and level were higher in testes than in ovaries. Hypermethylation in the gene bodies enhanced the expression of male-biased genes (such as DMRT1-SOX9-AMH and steroid hormone biosynthesis related genes) in the testis, as opposed to the normalization of gene expression. Our results provide insights into the genetic and epigenetic mechanisms underlying sex maintenance in adult Chinese alligators, and are expected to contribute to the development of scientific programs for the successful conservation of this endangered species.

## Introduction

The sex ratio in animal populations plays an important role in mate competition and mate selection, and thus affects species survival and population development (Dyson and Hurst, 2004). A pronounced bias in the sex ratio could potentially contribute to population collapse or even species extinction, particularly in small populations of endangered animals, the habitats of which are typically fragmented (Dale, 2001; Le Galliard et al., 2005). Accordingly, the mechanisms underlying sex determination and sex maintenance have been a particular focus of study for biologists.

The Chinese alligator (*Alligator sinensis*) is an endangered reptile that is currently endemic to the lower reaches of the Yangtze River (Chen et al., 2003; Pan et al., 2019). As in other crocodilian species, the sexes of Chinese alligator are fully determined by the temperature of eggs, the so-called temperature sensitive period (TSP), and remain irreversibly fixed thereafter (Lang and Andrews, 1994; Lin et al., 2018). This contrasts with events in sex reversal species, in which the sexes are determined by genes during the early phase of development, but can be reversed in response to certain environmental factors. Examples of sex reversal species include the half-smooth tongue sole (*Cynoglossus semilaevis*) (Shao et al., 2014), Nile tilapia (*Oreochromis niloticus*) (Sun et al., 2016), common frog (*Rana temporaria*) (Wallace et al., 1999), and Australian central bearded dragon lizard (*Pogona vitticeps*) (Quinn et al., 2007).

Most studies conducted to date on species characterized by temperature-dependent sex determination (TSD) have tended to focus on sex determination and differentiation during embryonic development, and comparatively little attention has been devoted to sex maintenance and gonadal function in adult individuals. Numerous genes involved in the progress of sex determination have been found to be conserved among TSD species and species characterized by genotypic sex determination (GSD). For example, male determination genes (e.g., *SOX9, SOX8, SOX10, AMH, DMRT1*, and *DAX1)* and female determination genes (e.g., *WNT4, RSPO1, FOXL2, and CYP19A*) have been shown to be involved in the determination of sex in TSD reptiles (Shoemaker and Crews, 2009). With the development of sequencing technology, RNA sequencing (RNA-Seq) of the embryonic gonads of several species, including the Chinese alligator (Lin et al., 2018), American alligator (*Alligator mississippiensis*) (Yatsu et al., 2016), red-eared slider turtle (*Trachemys scripta elegans*) (Czerwinski et al., 2016), and painted turtles (*Chrysemys picta*) (Radhakrishnan et al., 2017), has been conducted to examine the molecular mechanisms underlying the progression of TSD.

Recently, transcriptomic analyses have also been used in studies examining sex maintenance and gonadal function in adult organisms; however, most of these have tended to focus on invertebrates and fishes, such as the Nile tilapia and yellow catfish (*Pelteobagrus fulvidraco*), which are characterized by an XX/XY sex determination system (SDS) (Lu et al., 2014; Wang et al., 2016), Amur sturgeon (*Acipenser schrenckii*) with a ZZ/ZW SDS (Jin et al., 2015), spotted knifejaw (*Oplegnathus punctatus*) with an X_1_X_1_X_2_X_2_/X_1_X_2_Y SDS (Du et al., 2017), and Japanese mantis shrimp (*Oratosquilla oratoria*) and scallop (*Patinopecten yessoensis*), in which the SDSs have yet to be determined (Li et al., 2016; Yan et al., 2018). Comparison of the transcriptomes of gonads in adult Chinese pond turtles, the sex of which is fully determined by egg temperature, with expression level data (RT-qPCR) of selected TSD genes in embryonic gonads, has revealed that certain genes associated with sex determination and differentiation (*SOX9, AMH, AR* associated with males and *FOXL2, WNT4*, and *CYP19A* associated with females) exhibit similar expression patterns in embryonic and adult gonads, whereas others differ. For example, *DMRT1*, a conserved sex-determining gene in vertebrates encoding doublesex and mab-3 related transcription factor 1, shows a significant male-biased expression in embryonic gonads, whereas in adult testes, its expression is suppressed and shows similar expression levels in the ovary (Tang et al., 2017; Xiong et al., 2019). This suggests that *DMRT1* is crucial in male determination and differentiation during embryonic development, but less important for male sex maintenance and testis development in the adult turtle. Thus, to gain a better understanding of the molecular mechanisms underlying sex maintenance and gonadal function in adult individuals of the Chinese alligator, it would be of particular interest to characterize and compare the transcriptomes of adult and embryonic gonads in this TSD species.

DNA methylation is one of the most extensively studied epigenetic mechanisms and plays important roles in the regulation of gene expression, development, and stress responses (Smith and Meissner, 2013; Schubeler, 2015). The regulation of DNA methylation is notably complex, with the levels and roles of DNA methylation differing in different species and gene elements (Feng et al., 2010; Zemach and Zilberman, 2010; Zemach et al., 2010). In general, DNA methylation in gene promoters can alter chromatin structure and repress transcription, whereas high methylation levels in the gene body (introns and exons) are essential for the appropriate regulation of gene expression and alternative splicing (Yong et al., 2016). In this regard, methylation patterns in the half-smooth tongue sole have previously been determined using bisulfite sequencing (BS-Seq), and on the basis of these findings, it has been suggested that DNA methylation plays an important role in the sex reversal of fish (Shao et al., 2014). However, the roles of DNA methylation in the sex maintenance and gonadal function of adult individuals of TSD species have yet to be sufficiently determined.

In this study, we conducted RNA-Seq and BS-Seq analyses of the gonads of adult Chinese alligators to obtain high-resolution maps of the associated transcriptome and DNA methylome and to characterize the patterns of gene expression and DNA methylation. We identified key DNA modifications and the significant factors and essential pathways associated with sex maintenance and gonadal development, thereby providing valuable insights into the epigenetic mechanisms underlying sex maintenance and gonadal development in the Chinese alligator.

## Materials and Methods

### Sample Sources and DNA and RNA Extraction

The collection of samples for the purposes of this study was approved by the State Forestry Administration of the People’s Republic of China [Forest Conservation Permission Document (2014-1545)] and the Animal Ethics Committee of Zhejiang University (ZJU2015-154-13). All the samples were collected from the Changxing Yinjiabian Chinese Alligator Nature Reserve in 2015. Samples of winter-collected testis (WM_TES) and ovary (WF_OVA) were obtained from two hibernating adult Chinese alligators, whereas summer samples (SM_TES and SF_OVA) were collected from two adult alligators during the breeding season in May. All alligators were euthanized under deep anesthesia by injecting ketamine (5–10 mg/kg) into the tail muscle, prior to bloodletting performed in accordance with the guidelines of the Animal Ethics Committee of Zhejiang University. In addition to gonads, samples of other tissues were collected for a separate study (Lin et al., 2020a). Following collection, all samples were immediately frozen and then maintained in liquid nitrogen until use. gDNA and RNA were extracted from the testes and ovaries using an AllPrep DNA/RNA Mini Kit (Qiagen, Hilden, Germany), according to the manufacturer’s instructions.

### Strand-Specific cDNA Library Construction and Sequencing

For each sample, 3 mg of extracted RNA was used to construct strand-specific cDNA libraries using an NEBNext^®^ Ultra^TM^ Directional RNA Library Prep Kit for Illumina^®^ (New England Biolabs, Ipswich, MA), according to the manufacturer’s instructions. The index-coded samples were clustered using a TruSeq PE Cluster Kit v3-cBot-HS in a cBot Cluster Generation System (Illumina, San Diego, CA, United States), and the Illumina HiSeq 2500 platform was used to sequence the strand-specific cDNA libraries and generate raw data of 125 bp paired-end reads. In-house Perl scripts were used to remove reads containing adapters or poly N sequences and low quality reads, and the clean data were used in subsequent analyses.

### Bisulfite Library Construction and Sequencing

For each sample, 30 ng unmethylated λ-DNA was added to 6 mg gDNA and fragmented into 200–300-bp sequences *via* sonication. Subsequent to end repairing, acetylation, and the addition of barcodes containing methylated cytosines, we used an EZ DNA methylation-Gold^TM^ Kit (Zymo Research, Irvine, CA) to conduct two rounds of bisulfite conversion to ensure a high conversion rate. The conversion products were amplified by PCR using KAPA Hifi HotStart Uracil + ReadyMix (Kapa Biosystems, Wilmington, MA). The index-coded samples were clustered using a TruSeq PE Cluster Kit v3-cBot-HS in a cBot Cluster Generation System (Illumina), according to the manufacturer’s instructions. The Illumina HiSeq 2500 platform was used to sequence the bisulfite library and generate raw data of 125-bp paired-end reads. In-house Perl scripts were used to filter out low-quality reads, reads containing adapters or poly N sequences, and reads shorter than 36 nt following adapter removal, and the clean data were used in subsequent analyses. The results were visualized using GraphPad Prism 8.0.

### RNA Sequencing Data Analysis

An index of the Chinese alligator reference genome (Wan et al., 2013) was constructed using Bowtie2 (Langmead and Salzberg, 2012), and the clean reads were mapped to the Chinese alligator reference genome using TopHat v. 2.0.12 (Kim et al., 2013). The number of reads mapped to each gene was calculated using HTSeq v. 0.6.1 (Anders et al., 2015). Sex-biased differential gene expression was analyzed using the DEGseq R package v. 1.12.0 (Wang et al., 2010) based on stringent criteria [adjusted *p* (*q*) < 0.005 and | log2 (fold change)| > 1]. The FPKM (number of fragments per kilobase of exon per million mapped fragments) value of each gene was calculated to estimate gene expression levels.

### Bisulfite Sequencing Data Analysis

We generated a “T genome” (C-to-T converted) and an “A genome” (G-to-A converted) by converting the Chinese alligator reference genome *in silico* into fully bisulfite-converted versions and then indexed both using Bowtie2 (Langmead and Salzberg, 2012). Using default parameters, the C-to-T converted BS-seq reads were aligned to the “T genome,” and the G-to-A converted BS-Seq reads were aligned to the “A genome” using Bismark (v. 0.12.5) (Krueger and Andrews, 2011). The reads that produced a unique and best alignment from the two alignments were aligned back to the original Chinese alligator reference genome, and to infer the methylation state of each cytosine in the reads. Multiple reads mapped to the same genomic regions were removed to avoid inaccuracy, as these may have been derived from PCR duplicates. The bisulfite library conversion rate was estimated as the rate of thymines sequenced at cytosine positions in the non-methylated λ-DNA reference genome.

To identify methylated cytosines with strong statistical power, we modeled the summed mC of methylated counts as a binomial (Bin) random variable with methylation rate (r), as mC ∼ Bin (mC + umC^∗^r). The methylation level of each cytosine site was estimated as the number of reads containing an mC at the site of interest divided by the total number of reads covering the cytosine site. The methylation level of a genomic region was quantified as the average methylation level of all cytosines in this region. The results were visualized as scatter and violinbox plots using the R package ggplot2.

Sex-biased differentially methylated regions (DMRs) were identified using swDMR (Wang et al., 2015). We focused solely on DNA methylation in CG contexts, given that most of the identified methylated cytosines were in CG sites and the methylation levels in CHH and CHG contexts were extremely low. We set a sliding window of 1,000 bp with a step length of 100 bp. To ensure statistical power, we only considered those windows containing at least ten CG sites and were covered by at least five reads in each of the two compared samples. Data were analyzed using Fisher’s exact test, and only windows with an adjusted *p* (*q*) < 0.05 and a more than twofold change in methylation level were considered DMRs. Genes containing DMRs in their putative promoter and/or gene body were regarded as differentially methylated genes.

## Results

### Sex-Biased Expressed Genes in Adult Chinese Alligator Gonads

To determine the molecular mechanisms underlying sex maintenance and gonadal function development in adult Chinese alligators, we compared testis and ovary samples and identified the sex-biased differentially expressed mRNA-encoding genes (DEGs) using stringent criteria [*q* < 0.005 and | log2 (fold change)| > 1]. We identified the sex-biased DEGs separately in summer- and winter-collected samples, rather than treating these as biological replicates, as the physiological status of the alligators differs substantially in these two seasons (Lin et al., 2020a). In particular, given that the biosynthesis pathways of anti-Müllerian hormone, melatonin, and steroid hormones are suppressed in the testes during winter (Lin et al., 2020b), we focused on the summer-collected samples in the following analysis, as these provided a more representative indication of physiological status. However, we will refer to the winter-collected data in certain instances.

**FIGURE 1:**
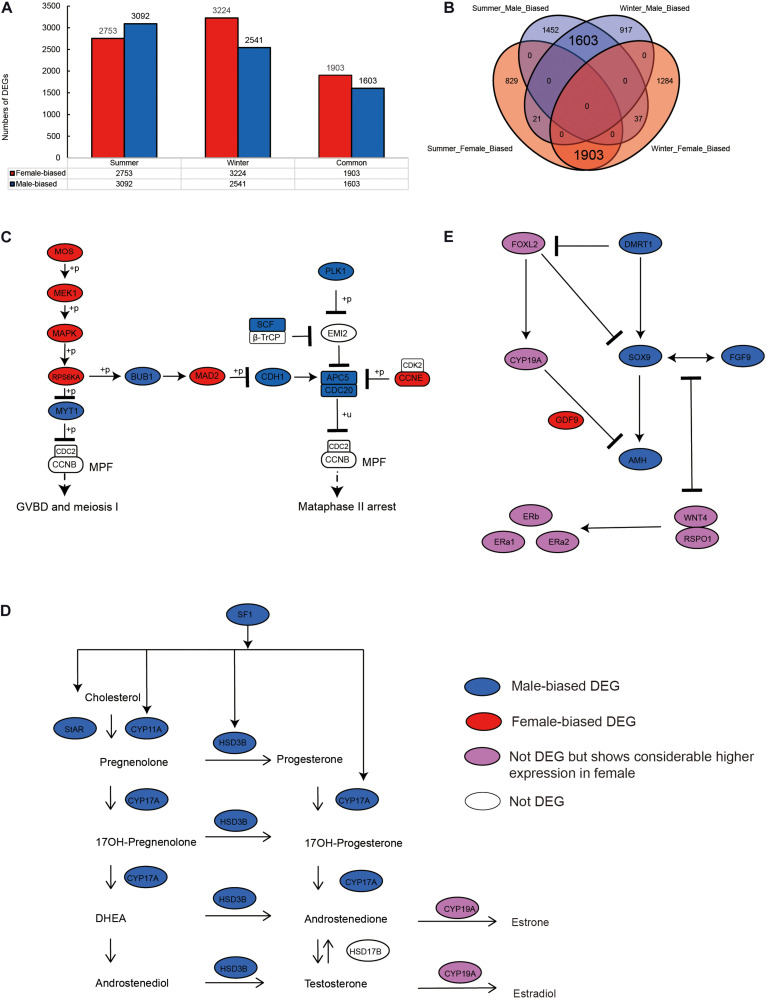
Sex-biased gene expression profiling in the gonads of adult Chinese alligators. **(A)** Numbers of sex-biased differentially expressed genes (DEGs) in gonads collected in summer and winter. **(B)** Overlap of female- and male-biased DEGs in gonads collected in summer and winter. **(C)** Expression pattern of genes in oocyte meiosis pathway. **(D)** Expression pattern of genes in steroid hormone biosynthesis pathway. **(E)** Expression pattern of genes in antagonistic pathways for maintenance and gonadal development.

We identified 5,845 sex-biased DEGs (comprising 3,092 male- and 2,753 female-biased DEGs) in summer, when the alligators were actively breeding (Figure 1A), and 5,765 sex-biased DEGs (including 2,541 male- and 3,224 female-biased DEGs) in hibernating alligators (Figure 1A). To eliminate the error introduced by sampling during different seasons, we merged the DEGs identified in summer and winter, and detected 3,564 genes showing sex-biased expression in both summer and winter, 3,506 (98.37%) of which showed the same sex-biased expression status in the two seasons (Figures 1A,B).

To identify genes and pathways that play important roles in sex maintenance and gonadal development, we compiled a list of 167 genes that are likely to participate in male determination/differentiation, female determination/differentiation, oocyte meiosis, and steroid hormone biosynthesis pathways (Supplementary Table 1), based on previous studies in vertebrate embryonic and adult gonads (Shoemaker and Crews, 2009; Eggers and Sinclair, 2012; Eggers et al., 2014; Windley and Wilhelm, 2015; Lin et al., 2018), and compared their expression levels in the ovary and testis.

Oocyte meiotic maturation is a process whereby immature oocytes become fertilizable eggs and is important for female breeding. Our results indicated that a range of different genes and signaling pathways are activated or suppressed to facilitate functional development in Chinese alligator ovaries during the breeding season. In particular, genes in the MOS-MEK1-MAPK-PSK pathways were overexpressed, whereas *MYT1* was suppressed, thus stimulating the resumption of meiosis *via* activation of the maturation promoting factor (MPF). *APC5, CDC20, CDH1, SCF*, and *PLK1*, the suppression of which is required for the reactivation of MPF for meiosis II, were downregulated in the ovary, whereas *MAD2* and *CCNE*, factors associated with the inhibition of these genes, were overexpressed (Figure 1C and Supplementary Table 1). We found that the sex-biased gene expression pattern in winter was similar to that in summer (Supplementary Table 1), thereby indicating that the ovary continues to develop rapidly during hibernation rather than slowing, as in other tissues (Lin et al., 2020a).

During embryonic ovary development, steroid hormone biosynthesis pathway genes are highly expressed, thus indicating the essential role of steroid hormones in female sex determination (Lin et al., 2018). However, we detected different patterns in adult alligator gonads. Genes associated with steroid hormone biosynthesis (*CYP11A, CYP17A*, and *HSD3B*) and their regulatory factor *SF1*, as well as *STAR*, which encoding the rate-limiting enzyme steroidogenic acute regulatory protein, showed higher expression levels in the testis (Figure 1D and Supplementary Table 1). This indicates the important roles of androgenic hormones in male sex maintenance and testis development. In adult ovary, most steroid hormone biosynthesis pathway genes were suppressed. However, *CYP19A*, which encodes the cytochrome P450 aromatase that irreversibly converts androgens to estrogens (Figure 1D and Supplementary Table 1), as well as its upstream regulator *FOXL2*, and estrogen receptor genes (*ERa1, ERa2*, and *ERb*) was found to be expressed at considerably higher levels in the ovary (Figure 1E and Supplementary Table 1). This results suggest that estrogens are still crucial for female sex maintenance and ovary development, although in most instances, the differences in expression were not statistically significant.

Several male sex determination/differentiation genes, including *DMRT1, SOX9, FGF9*, and *AMH*, were also observed to be highly expressed in the testis, constituting a pathway (and positive feedback loop) for male sex maintenance. In contrast, the expression levels of female sex determination/differentiation genes, including *GDF9, RSPO1*, and *WNT4B*, were found to be considerably higher in the ovary than in the testis (Figure 1E and Supplementary Table 1).

Overall, we identified the genes and pathways involved in sex maintenance and gonadal function development in adult Chinese alligators, among which, the expression patterns of certain pathways were found to be similar to those in embryos of the Chinese alligator and other species, whereas others are unique to adult Chinese alligators.

### Higher Methylation Levels in Testes Than in Ovaries

**FIGURE 2:**
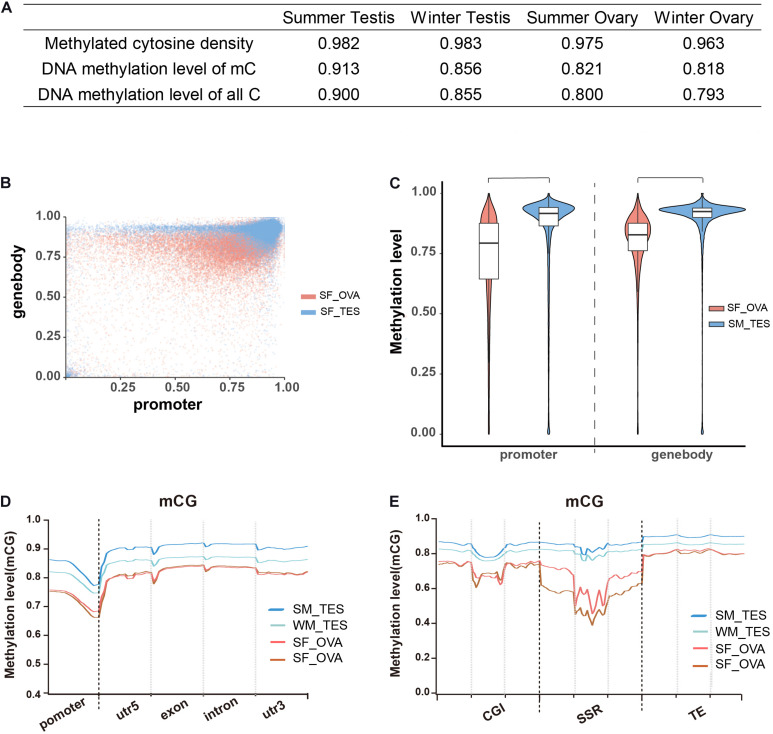
Overall DNA methylation patterns of CG context in adult Chinese alligator gonads. **(A)** The DNA methylation density and level of gonad samples. **(B)** Methylation levels of genes in the ovary and testis collected in summer. **(C)** Violin plot of the methylation levels of genes in the ovary and testis collected in summer. **(D)** DNA methylation levels in different elements of genes in the gonads of adult Chinese alligators. Promoters encompass the 2-kb region upstream of the transcription start site. **(E)** DNA methylation levels in CG islands (CGI), simple-sequence repeats (SSR), and transposable elements (TE) and 2-kb regions upstream and downstream of genes in the gonads of adult Chinese alligators.

To examine the roles of DNA methylation in sex maintenance and gonadal function development in adult Chinese alligators, we compared DNA methylation patterns in the testes and ovaries. We accordingly found that not only the densities of methylated cytosine but also their average methylation levels were higher in testes than in ovaries (Figure 2A). Calculating the DNA methylation levels of each gene in the testes revealed that gene methylation levels were more clustered at high levels, particularly those in gene bodies, whereas levels in the ovaries were more diverse, with a considerably larger number of genes exhibiting lower methylation (Figure 2B). Regardless of distribution within the gene sequence, overall gene methylation levels were significantly higher in the testis than in the ovary (Wilcox test, *p* < 0.05) (Figures 2C,D). Moreover, methylation levels in GC islands, microsatellites, transposable elements, and adjacent regions were also shown to be higher in the testis (Figure 2E).

Comparison of whole-genome DNA methylation patterns in the testes and ovaries of the Chinese alligator revealed sex-biased DMRs in the genomes. We identified 39,335 DMRs (43,268,052-bp long) in the summer-collected gonadal samples, among which 18,855 (47.93%) overlapped with the promoter and/or gene body of 10,587 genes (so-called differentially methylated genes, DMGs). Consistent with previous results, we identified a considerably larger number of testis-hypermethylated-genes (10,052) than testis-hypomethylated-genes (843). Furthermore, most gene-linked DMRs (16,334, 86.61%) were found to be distributed in gene bodies, with only a few extending to the promoter region. The winter-collected gonadal samples showed similar patterns. Collectively, these results indicate that hypermethylation in gene bodies plays an important role in male sex maintenance and testis functional development.

### Hypermethylation Enhances Gene Expression in the Testis

**FIGURE 3:**
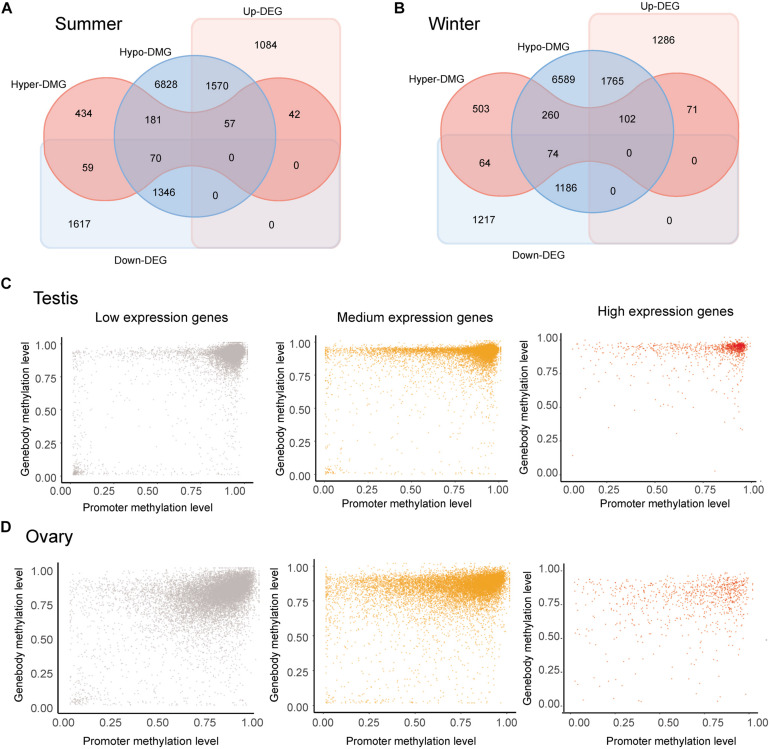
Correlation analysis of the methylome and transcriptome. **(A,B)** Venn diagram of sex-biased differentially methylated genes (DMGs) and differentially expressed genes (DEGs) in gonads collected in the summer **(A)** and winter **(B)**. **(C,D)** Methylation levels in the promoter and gene body of genes showing low (FPKM < 1), medium (1 < FPKM < 100), and high (FPKM > 100) expression in the ovary **(C)** and testis **(D)** collected in summer.

The occurrence of overlapping DMGs and DEGs revealed that genes with sex-biased DMRs were significantly more likely to be sex-differentially expressed (Pearson’s chi-square test, *p* < 0.05) (Figures 3A,B). Methylation patterns differed in genes showing different levels of expression levels. Regardless of expression level, however, DNA methylation levels were generally extremely high in the gene bodies of most genes. Nevertheless, whereas DNA methylation levels tend to be high in the promoter regions of most lowly expressed genes, they were found to be lower in genes showing medium/high expression, thereby indicating a negative correlation between the levels of promoter DNA methylation and gene expression (Figure 3C). Although we observed similar overall methylation patterns in testes and ovaries, methylation in the former was found to be more clustered at higher levels (Figure 3D).

**FIGURE 4:**
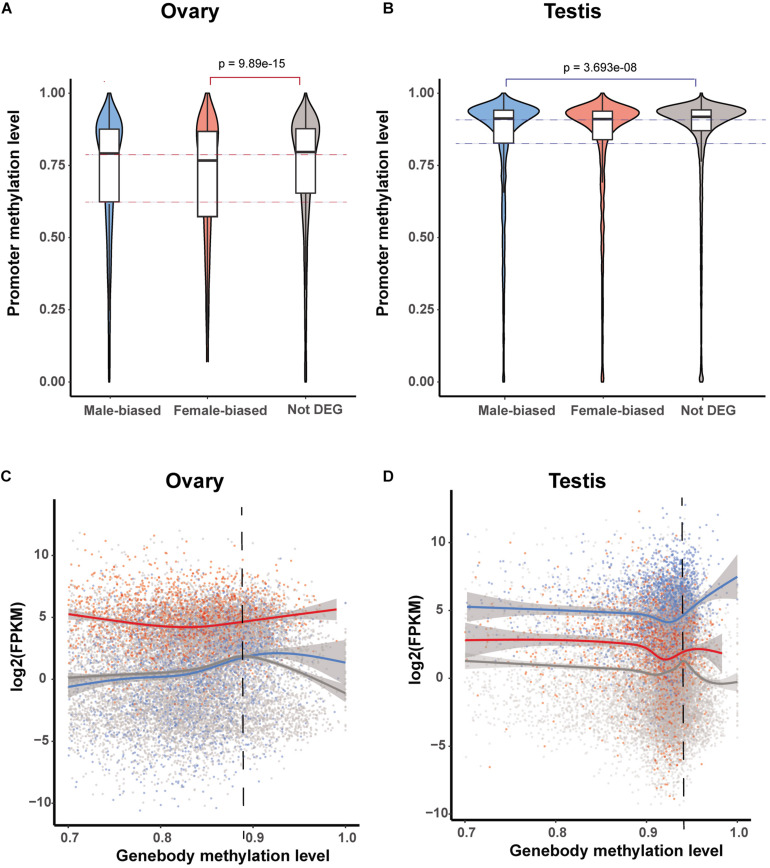
Methylation patterns of sex-biased differentially expressed genes (DEGs). **(A,B)** Violin plot of methylation levels of male- and female-biased DEGs and non-DEGs among genes in the ovary **(A)** and testis **(B)**. **(C,D)** Correlation between gene expression level and gene body methylation level for male- and female-biased DEGs and non-DEGs among genes in the ovary **(C)** and testis **(D)**.

To further investigate the roles of promoter and gene body methylation in gene expression regulation, we compared DNA methylation levels in both of these gene elements in those genes showing sex-biased expression in the testis and ovary. In the ovary, the methylation level of the promoters in female-biased DEGs was found to be significantly lower than that of non-biased DEGs (Wilcox test, *p* < 0.05) (Figure 4A). Similarly, in the testis, the methylation level of the promoters in male-biased DEGs was significantly lower than that of non-biased DEGs (Wilcox test, *p* < 0.05) (Figure 4B). These observations again tend to indicate that low levels of promoter methylation are correlated with active gene expression in gonads.

It has previously been reported that gene-body methylation is associated with the normalization of gene overexpression (Yong et al., 2016; Lin et al., 2020a). Consistently, we detected a similar pattern among genes in the ovary as well as in female-biased and non-biased DEGs in the testis (Figures 4A–C), however, unexpectedly, this was not observed in male-biased DEGs in the testis. Among the genes with high methylation levels, the expression level of male-biased DEGs was positively correlated with the level of methylation (Figure 4D).

**FIGURE 5:**
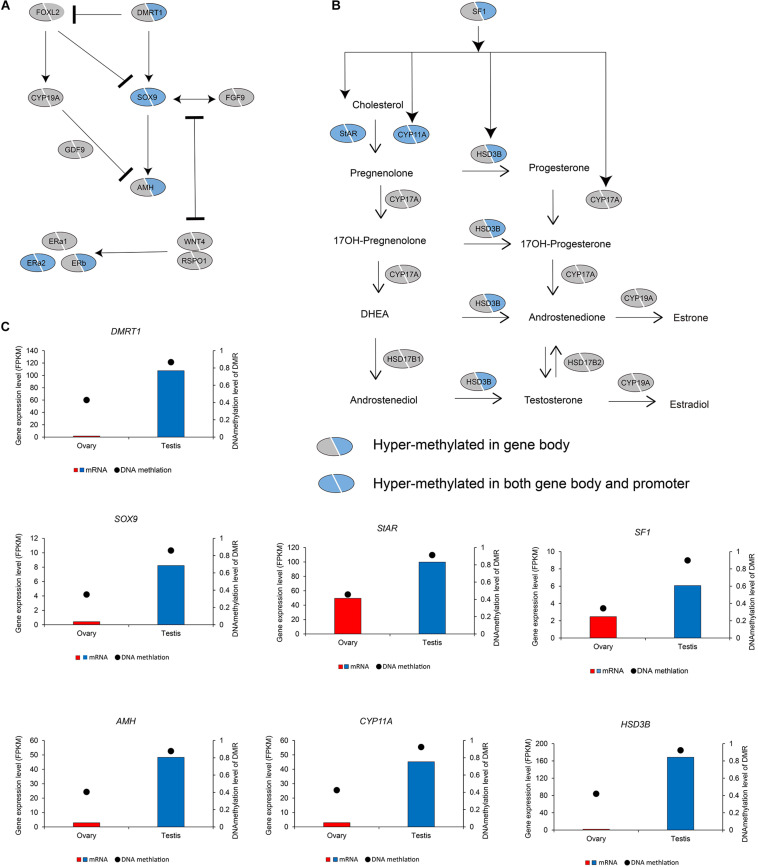
DNA methylation patterns of genes in antagonistic pathways for sex maintenance and gonadal development **(A)** and steroid hormone biosynthesis pathway **(B)**, as well as DNA methylation levels of differentially methylated regions (DMRs) and the expression levels of key genes in these pathways **(C)**.

**FIGURE 6:**
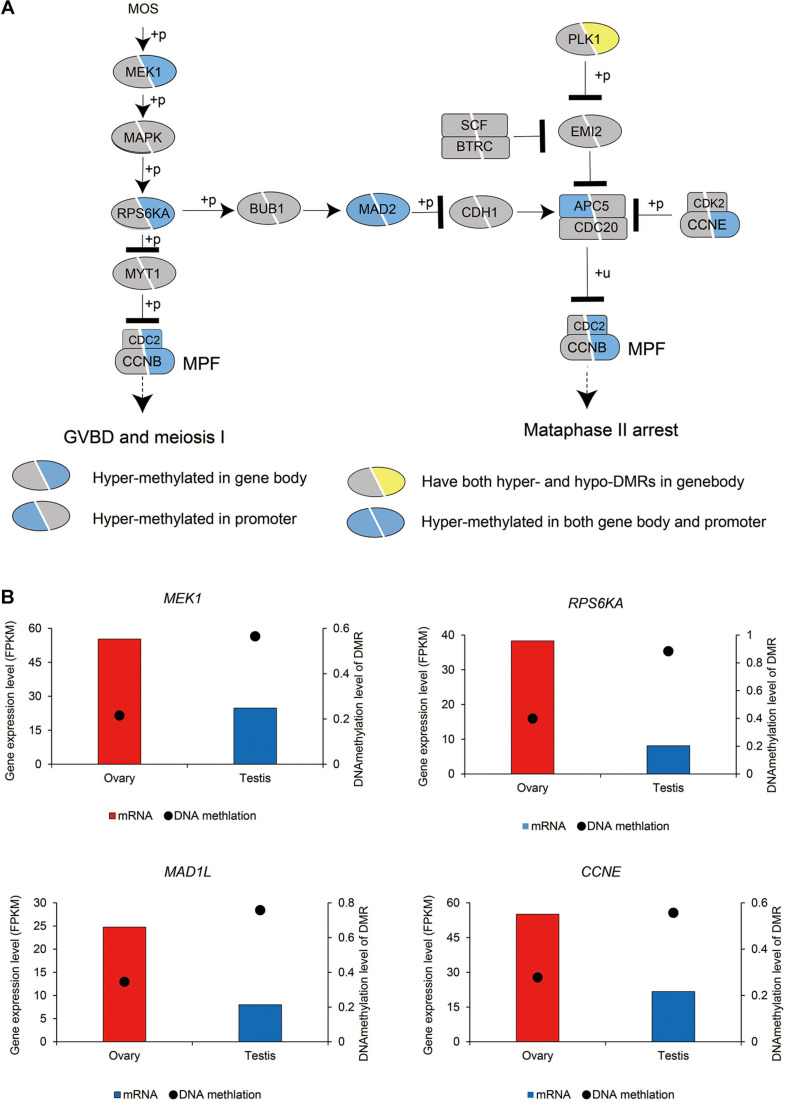
DNA methylation patterns of genes in the oocyte meiosis pathway **(A)** and DNA methylation levels of differentially methylated regions (DMRs) and the expression levels of key genes in this pathway **(B)**.

We subsequently investigated the methylation status of 167 genes in sex maintenance and gonadal development pathways, and, in line with expectations, we identified 92 (55.08%) DMGs, among which 86 were hypermethylated (Supplementary Table 2). In particular, we detected hypermethylation in the gene bodies of the male determination genes *DMRT1, SOX9*, and *AMH* and four steroid hormone biosynthesis pathway genes, *SF1, STAR, CYP11A*, and *HSD3B* (Figure 5). These results thus tend to indicate that in the testis, DNA methylation in the gene bodies of male-biased genes does not normalize gene overexpression, but instead enhances gene expression, as these highly expressed genes are considered important in testis development and spermatogenesis in adult Chinese alligators. However, the role of DNA methylation in the oocyte meiotic maturation pathway appears to be considerably more complex. Four female-biased DEGs (*MEK1, RPS6KA, MAD2*, and *CCNE*) in this pathway were found to hypomethylated in the ovary, whereas we detected no sex-biased DMRs in the male-biased genes *MYT1, BUB1, CDH1, SCF, APC5*, and *CDC20* (Figure 6).

## Discussion

Having been determined in the temperature-sensitive period (TSP) during embryonic development, the sex of Chinese alligators remains fixed thereafter. In this study, we performed RNA-Seq and BS-Seq analyses to determine the genetic and epigenetic mechanisms underlying sex maintenance and gonadal development in adult Chinese alligators.

To date, most research that has been conducted on TSD species has tended to focus on sex determination and differentiation during embryonic development, whereas comparatively less attention has been devoted to sex maintenance and gonadal development in adult individuals, particularly among those studies using high-throughput technology. In the gonads of adult Chinese pond turtles, some genes in sex determination and differentiation pathways (*SOX9, AMH, AR, FOXL2, WNT4*, and *CYP19A*) exhibit similar expression patterns in embryonic and adult gonads. In contrast, whereas *DMRT1* shows a significantly male-biased expression in embryonic gonads, its expression in the testes of adult alligators is suppressed, showing expression levels similar to those observed in the adult ovary (Tang et al., 2017; Xiong et al., 2019).

Compared with our previous transcriptome study on embryonic sex determination in the Chinese alligator (Lin et al., 2018), we also identified several genes showing similar sex-biased patterns in embryonic and adult individuals. In the embryos of Chinese alligator and mammals, SOX9 (SRY-box transcription factor 9) plays a central role during male determination and differentiation by activating the expression of anti-Müllerian hormone gene *AMH*, and forming a positive feedback loop with FGF9 (fibroblast growth factor 9) (Figure 1E; Shoemaker and Crews, 2009). The male-biased expression of *SOX9, AMH*, and *FGF9* in adult alligator gonads suggests their conserved roles across testis development of Chinese alligator. In addition, although not statistically significant, *AR* encoding androgen receptor is expressed at a higher in testis. On the other hand, *RSPO1* (R-spondin 1) and *WNT4* (wingless integration site family member 4) have been reported to antagonize the *SOX9*/*FGF9* male determining/differentiation positive feedback loop (Figure 1E; Kim et al., 2006; Tomizuka et al., 2008), suggesting the persistent existence of this female-male antagonism pathway.

In contrast to patterns observed in the Chinese pond turtle, we found that the expression levels of *DMRT1* were low and identical in the embryonic gonads of both male and female alligators, and that this gene is actively expressed in adult alligator testes, thereby indicating its temporal role in the sex maintenance and gonadal development of adult Chinese alligators and its diverse functions in this reptile. This is consistent with previous a report that *DMRT1* is essential to maintain mammalian testis determination; XY mice that have loss *DMRT1* are born as males with testes, but the gonads later undergo abnormal differentiation (Matson et al., 2011).

Several lines of evidence indicate that in addition to sex change in sex reversal species (Nakamura et al., 1989), steroid hormones play important roles not only in the sex determination of GSD and TSD species (Crews, 1996; Morohashi et al., 2013) but also in the development of sexual dimorphism (Morohashi et al., 2013). With the progression of male sex determination in Chinese alligator embryos, there is a decline in the activity of steroid hormone biosynthesis pathways at high temperatures associated with male determination, thus indicating that steroid hormone genes play a more important role in fetal female determination (Lin et al., 2018). However, the findings of the current study revealed a contrasting expression pattern. The expression of steroid hormone biosynthesis genes, including *STAR* (the rate-limiting factor of this pathway), were found to be male-biased rather than female-biased, which tends to indicate that steroid hormones play a more important role in male sex maintenance and testis function development.

DNA methylation, the most extensively studied epigenetic modification, plays an important role in gonad development. For example, in the half-smooth tongue sole, with the exception of the W chromosome, overall methylation levels are higher in the testis of ZZ males and high-temperature-induced pseudo males (ZW) than in the ovary (Shao et al., 2014). In the Nile tilapia, another fish characterized as a temperature-dependent sex reversal, although the overall methylation levels in the normal testis (XY) are lower than those in the normal ovary (XX), high-temperature notably enhances the methylation in the high-temperature-induced pseudo males (XX), which is even more methylated than those in high temperature-treated females (XX) (Sun et al., 2016). At the gene level, DNA methylation contributes to the regulation of gene expression and oocyte growth and sex differentiation (Nishino et al., 2004; Pierron et al., 2014). However, although several studies have examined the roles of DNA methylation in specific genes during sex determination of TSD species (Navarro-Martín et al., 2011; Matsumoto et al., 2013; Parrott et al., 2014), few studies, particularly those based on analysis using the so-called gold standard technology BS-Seq, have investigated the genome-wide DNA methylation pattern in adult gonads of TSD species. In the present study, we characterized at high resolution the genome-wide sexually dimorphic DNA methylation patterns in adult Chinese alligators and accordingly revealed that this reptile shows a pattern similar to that of the two fish species described above. In line with expectations, we found that both the density and level of DNA methylation were higher in testes than in ovaries, and that most DMRs in the testis were hypermethylated. By combining transcriptomes and methylomes, we identified several DMGs associated with male sex determination/differentiation, steroid hormone biosynthesis, and oocyte meiosis pathways. As the male sex fate of Chinese alligator was determined by high incubation temperature in TSP during embryonic development, we hypothesize that hypermethylation, in Chinese alligator, half-smooth tongue sole and Nile tilapia, is induced by high incubation temperate and then plays an important role in male sex maintenance and gonad development thereafter.

It has been established that the patterns and functions of DNA methylation differ in different genome elements (Feng et al., 2010; Zemach and Zilberman, 2010; Zemach et al., 2010), for example, promoter methylation being negatively correlated with gene expression. Contrarily, gene body methylation plays an important role in normalizing gene overexpression (Xu et al., 2018; Lin et al., 2020a). However, when we classified genes into three groups, namely, male-biased, female-biased, and non-DEGs, we unexpectedly discovered a different expression–methylation correlation in the male-biased DEGs in testis, particularly those with the highest DNA methylation levels, compared with the other two groups in the testis and all three groups in the ovary. In this regard, DNA methylation acts as an enhancer rather than a repressor, given that these genes (including *FGF9, SF1, STAR, CYP11A*, and *CYP17A*) play essential roles in testis development.

Our study would have benefited from using more biological replicates to enhance the validity of the findings. However, the Chinese alligator is under national first-class protection in China. Because of policies of the Chinese government and conservation guidelines, we used as few alligator individuals as possible in this study. To compensate for this limitation, we used software packages (DEGseq and swDMR) suitable for analyzing data lacking biological replicates for the identification of DEGs and DMRs (Wang et al., 2010) (Wang et al., 2015), using stringent criteria. Genes with *q* < 0.005 and | log2 (fold change)| > 1 were considered DEGs. For BS-Seq, to ensure proper statistical power, only windows with at least 10 CG sites and a coverage of five in each of the two compared samples were considered. Fisher’s exact test was employed and only windows with *q* < 0.05 and a greater than twofold methylation level change were considered DMRs. These criteria are equal to or even more stringent than those used in previous studies lacking biological replicates (Zhang et al., 2015; Li et al., 2017; Ma et al., 2019) to minimize false positives.

Collectively, the findings of this study provide insights into the genetic and epigenetic mechanisms underlying sex maintenance and gonadal function development in adult Chinese alligators. We anticipate that our findings will contribute to the development of scientific programs for the successful conservation of this endangered species.

## Data Availability Statement

Publicly available datasets were analyzed in this study. This data can be found here: The Chinese alligator reference genome is available from GenBank (assembly accession: GCA_000455745.1). The BS-Seq and RNA-Seq data used in this work have been deposited in the NCBI SRA database under BioProject accession numbers PRJNA556094 and PRJNA556093, respectively.

## Ethics Statement

The animal study was reviewed and approved by the Animal Ethics Committee of Zhejiang University (ZJU2015-154-13).

## Author Contributions

S-GF conceived, designed, and supervised the project. J-QL and LS collected the samples. J-QL extracted the DNA samples. J-QL and JY analyzed the data and drafted the manuscript. S-GF and J-QL revised the manuscript. All authors read and approved the final manuscript.

## Conflict of Interest

The authors declare that the research was conducted in the absence of any commercial or financial relationships that could be construed as a potential conflict of interest.

## Suppelementary Material

Supplementary Table 1 Expression patterns of genes involved in sex maintenance and gonadal function development.

Supplementary Table 2 Differentially methylated genes (DMGs) and/or differentially expressed genes (DEGs) between adult Chinese alligator gonads in pathways involved in sex maintenance and gonadal function development.
